# An in-line Mach-Zehnder Interferometer Using Thin-core Fiber for Ammonia Gas Sensing With High Sensitivity

**DOI:** 10.1038/srep44994

**Published:** 2017-04-05

**Authors:** Xinyue Huang, Xueming Li, Jianchun Yang, Chuanyi Tao, Xiaogang Guo, Hebin Bao, Yanjun Yin, Huifei Chen, Yuhua Zhu

**Affiliations:** 1 College of Chemistry and Chemical Engineering, Chongqing University, Chongqing, 400044, China; 2 College of Materials Science and Engineering, Chongqing University of Technology, Chongqing, 400044, China; 3 College of Optoelectronic Engineering, Chongqing University, Chongqing, 400044, China

## Abstract

Ammonia is an important indicator among environmental monitoring parameters. In this work, thin-core fiber Mach-Zehnder interferometer deposited with poly (acrylic acid) (PAA), poly (allyamine hydrochloride) (PAH) and single-walled carbon nanotubes (SWCNTs-COOH) sensing film for the detection of ammonia gas has been presented. The thin-core fiber modal interferometer was made by fusion splicing a small section of thin-core fiber (TCF) between two standard single mode fibers (SMF). A beam propagation method (BPM) is employed for the design of proposed interferometer and numerical simulation. Based on the simulation results, interferometer with a length of 2 cm of thin-core fiber is fabricated and experimentally studied. (PAH/PAA)_2_ + [PAH/(PAA + SWCNTs-COOH)]_8_ film is deposited on the outer surface of thin-core fiber via layer-by-layer (LbL) self-assembly technique. The gas sensor coated with (PAH/PAA)_2_ + [PAH/(PAA + SWCNTs-COOH)]_8_ film towards NH_3_ gas exposure at concentrations range from 1 to 960 ppm are analyzed and the sensing capability is demonstrated by optical spectrum analyzer (OSA). Experimental results show that the characteristic wavelength shift has an approximately linear relationship in the range 1–20 ppm, which is in accordance with the numerical simulation. Thus, this paper reveals the potential application of this sensor in monitoring low concentration NH_3_ gas.

As a new sensing technology, optical fiber sensor has shown a strong vitality in many areas, such as bio-medicine, chemistry, oil, aerospace and marine. Chemicals and reagents would cause health hazard to human body due to the strong corrosivity and toxicity^1^,^2^. Conventional sensors are not suitable for application in such extreme environment due to easy to rust and conduct. Compared to traditional sensor systems, optical fiber sensing systems have the extreme electrical safety features. Up to now, there are many reports about optic fiber chemical sensor, such as gas sensors^3^,^4^, liquid pH sensors^5^, organic liquid refractive index sensors^6^, humidity sensors^7^, etc.

Among various kinds of optical fiber sensors, those based on the interference theory account for a large proportion. Optical fiber interferometer mainly including: Michelson interferometer^8^,^9^, Mach-Zehnder interferometer^10^,^11^, Fabry-Perot interferometer^12^,^13^ and Sagnac fiber loop interferometer^14^,^15^. Mach-Zehnder interferometer is based on double beam interference structure with advantages of high precision, low cost, and easy to adjust, which is not only widely used in the distributed sensing^16^, but also applied to point measurement sensor^17^,^18^. In 1979, optical fiber pressure and temperature sensor based on the Mach-Zehnder interference was first proposed by G. B. Hocker^19^. The numerical calculation and experimental results had a good consistency. Until 1995 the interferometer based on the thin-core optical fiber was put forward by K. Watanabe, which was used for the measurement of displacement and liquid^20^. Subsequently, more and more interferometer fabricated with thin-core fiber and multimode fiber is applied to measure the pH value^21^,^22^, relative humidity^23^,^24^, temperature^25^,^26^, pressure^27^, refractive index^28^,^29^ and so on. While light sources and interference information receivers (spectrometer, photoelectric detector, etc.) are single mode fiber generally, the transmission mode in multimode fiber is complex, and the interference spectrum is irregular and hard to analysis. Single-mode optical fiber could realize single pattern transmission, so we choose single-mode fiber instead of multimode optical fiber to fabricate the Mach-Zehnder interferometer.

In this paper a comprehensive theoretical model for the thin-core fiber Mach-Zehnder interferometer is provided and simulations are carried out based on beam propagation method to the optimal design and investigate the performance of the interferometer. The interferometer consists of (PAH/PAA)_2_ + [PAH/(PAA + SWCNTs-COOH)]_8_ film-coated thin-core optical fiber. In order to further elucidate the sensing property, the dynamic responses and selectivity of interferometer were investigated. Experimental investigations are consistent well with simulation result.

## Results

### Numerical simulation

**Figure 1:**
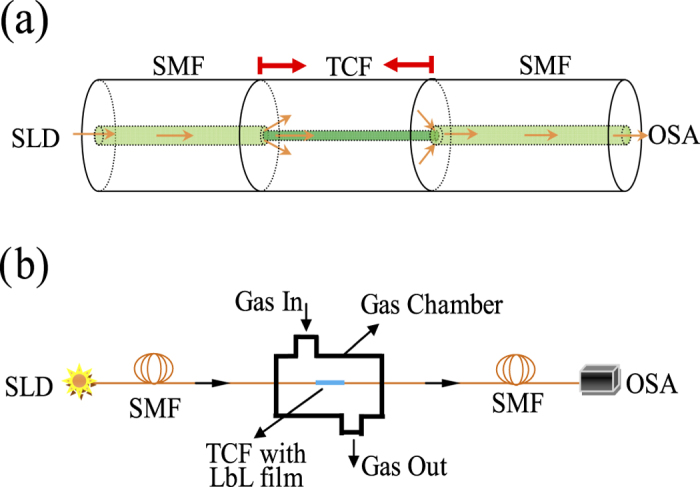
(**a**) Configuration of thin-core fiber Mach-Zehnder interferometer; (**b**) Schematic diagram of the experimental system.

The configuration of the thin-core fiber Mach-Zehnder interferometer is schematically shown in Fig. 1a. As shown in Fig. 1, both SMF and TCF have a step index profile and standard SMF are used as the connecting fiber at two ends of the thin-core fiber. When the light transmitted from the SMF into the TCF will excite cladding modes propagating within the cladding of the interferometer due to the core diameter of SMF is larger than TCF. Interference occurring within the cladding of the TCFMI will indicate the spectral response to the output SMF, which in turn can be affected by the RI of the external environment. By monitoring the spectral changes of the output of TCFMI, the RI of the external environment can be determined. Figure 1b has shown the schematic diagram of experimental setup for investigating the sensing capability of the thin-core fiber Mach-Zehnder interferometer sensor.

**Figure 2:**
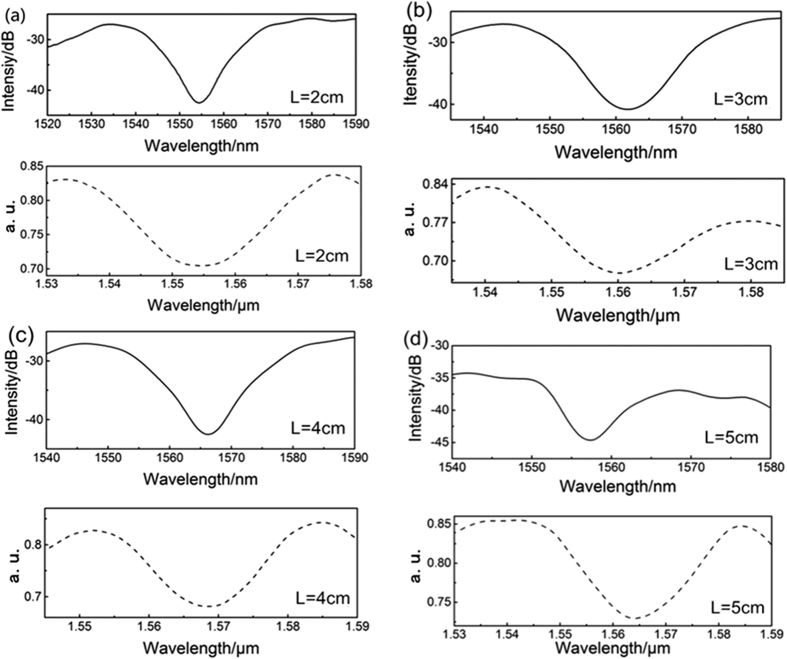
The experimental(solid line) and the simulative (dashed line) spectrum of thin-core fiber Mach-Zehnder interferometer with different length of thin-core fiber (**a**) 2 cm; (**b**) 3 cm; (**c**) 4 cm; (**d**) 5 cm.

**Figure 3:**
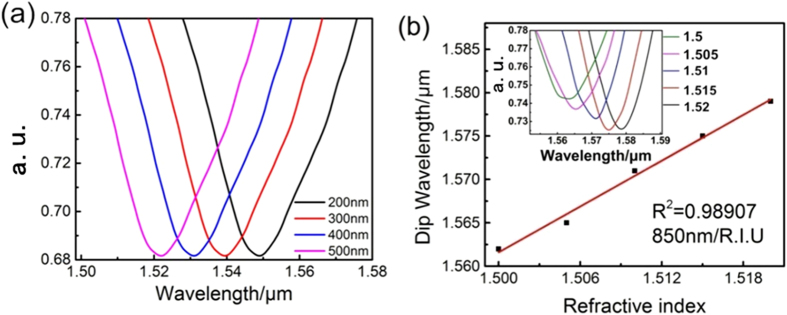
(**a**) The simulated spectrum of thin-core fiber Mach-Zehnder interferometer self-assembled with different thickness of film; (**b**) The simulated spectrum of thin-core fiber Mach-Zehnder interferometer in different refractive index solution.

In order to investigate the dependence of the interference fringe on the length of TCF, thin-core fiber Mach-Zehnder interferometer with different TCF lengths were simulated and fabricated. The optical fiber parameters selected in this numerical simulation are shown in Table 1. From Fig. 2, the experimental measured transmission spectrums are presented with solid lines, the simulation spectrums are labeled with dashed lines. The length of TCF in simulation and testing are 2,3,4,5 cm respectively. In this work, no matter how to change the length of TCF, the only one interference dip is formed in the wavelength range of 1520–1590 nm. The different lengths of the embedded thin-core fiber will form similar interference fringe but distinct positions of single transmission dip. In contrast, the experimental measured spectrums are well consistent with the simulated one throughout the entire transmission spectrum. Considering cost and fabrication, we have chosen the length of 2 cm thin-core fiber to prepare the thin-core fiber Mach-Zehnder interferometer in this work. Further simulations of central wavelength shift versus thickness and IR of film at length of 2 cm for TCF are carried out (Fig. 3). The simulated results in Fig. 3a confirm that the central wavelength of the spectral peak blue shift monotonically when increasing the thickness of film from 200 to 500 nm. Figure 3b shows the wavelength shift with different surrounding refraction index (SRI). Notably, from the inset of Fig. 3b the central wavelength of the spectral peak shifted toward the shorter wavelength direction with the RI decrease and the cumulative shift reached up to16 nm. Data in the inset of Fig. 3b is fitted by a linear regression model using the Origin Lab software with the correlation coefficient is 0.98907, indicating the linear response of the thin-core fiber Mach-Zehnder interferometer in the RI range. The RI theoretical sensitivity is 850 nm/R.I.U.

### Structural and morphological characterization

To evaluate the sensing performance of the thin-core fiber Mach-Zehnder interferometer NH_3_ gas sensors, the sensor coated with (PAH/PAA)_2_ + [PAH/(PAA + SWCNTs-COOH)]_8_ film is fabricated by Lay-by-Layer electrostatic self-assembly (EAS) technique. The ESA technique has advantages of simplicity in preparation and abundance in film-forming materials. It unacted on the shape and size of substrate, so it is feasible to deposit azimuthally symmetric coatings onto the cylindrical surface of thin-core optical fiber.

SWCNTs-COOH with nanoscale, large specific surface area and high length-diameter ratio, are easy to agglomerate and entangle due to high electrostatic interaction and van der Waals forces. Commercial SWCNTs-COOH is seriously agglomerated, resulting in the decrease of the electrical and mechanic properties. The addition of polymer into SWCNTs-COOH is an effective way to achieve dispersion.

**Figure 4:**
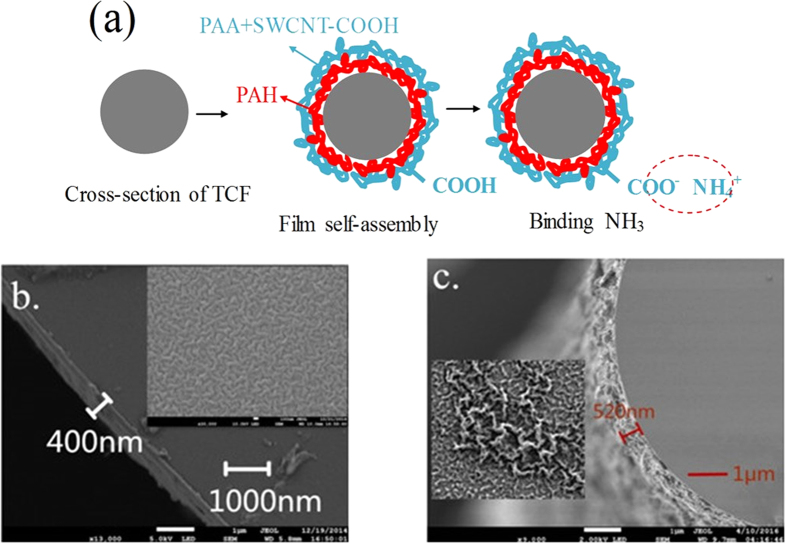
(**a**) Cross-sectional view of the TCF section. (**b**) The scanning electronic microscope (SEM) image of the cross-section of the fiber coated with (PAA/PAH)_10_ film. The inset shows the surface morphology. (**c**) The scanning electronic microscope (SEM) image of the cross-section of the fiber coated with (PAH/PAA)_2_ + [PAH/(PAA + SWCNTs-COOH)]_8_ film. The inset shows the surface morphology.

The operation of the sensor is based on the acid-base interaction of NH3 with carboxylic acid groups of PAA + SWCNTs-COOH hybrid system. PAA + SWCNTs-COOH hybrid system is a promising candidate for the fabrication of ammonia sensor on a variety of platforms due to the presence of free carboxylic acid functional groups and large surface area, which leads to high adsorption and selectivity toward amine compounds (The sketch for sensing mechanism is shown in Fig. 4a). In order to further observe the film on the surface of sensor, the cross-section of the TCF coated with the (PAA/PAH)_10_ and (PAH/PAA)_2_ + [PAH/(PAA + SWCNTs-COOH)]_8_ films are investigated by scanning electronic microscope (SEM) as shown in Fig. 4b and c. The (PAA/PAH)_2_ + [PAH/(PAA + SWCNTs-COOH)]_8_ film thickness is about 520 nm. The (PAA/PAH)_10_ film thickness is about 400 nm. It is found that the PAH/PAA films have average thicknesses of 40 nm. Consequently, we can predict the thickness of PAH/(PAA + SWCNTs-COOH) layers is about 55 nm and the thickness of SWCNTs-COOH is about 15 nm. Compare the Fig. 4b and c, the PAH/PAA film is dense and homogeneous. After adding SWCNTs-COOH into the PAA solution, the PAH/(PAA + SWCNTs-COOH) film become more rough and fluffy. From the cross-section image of the TCF coated with the PAH/(PAA + SWCNTs-COOH), there exist many gaps in the filamentous structure. Therefore the existence of SWCNTs-COOH increase the specific surface area of the film and make the ammonia gas fully contact with the film.

### Sensing performance of the TCFMI sensor

The as-prepared thin-core fiber Mach-Zehnder interferometer sensor coated with (PAH/PAA)_2_ + [PAH/(PAA + SWCNTs-COOH)]_8_ film was tested with different concentration of ammonia gas. Ammonia and nitrogen are diffused by two channels respectively, through the gas flow controller blend into different concentration of mixed gas. The sensitive region was attached to a gas chamber in order to avoid undesired bending during all the measuring processes.

**Figure 5:**
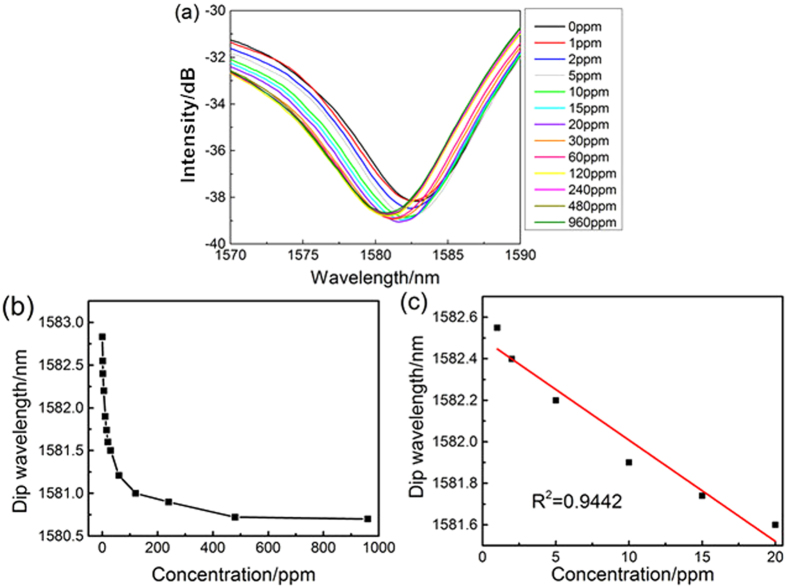
(**a**) The spectral responses of the thin-core fiber Mach-Zehnder interferometer sensor in various concentration of ammonia gas. (**b**) The wavelength shift upon the concentration of ammonia gas (0–960 ppm); (**c**) The wavelength shift upon the concentration of ammonia gas (0–20 ppm).

The transmission spectrums of ammonia with different concentrations were recorded by the OSA (Fig. 5a). To regenerate the sensor response, the optical fiber was treated with pure N_2_ during every interval of detection. Figure 5b shows the spectral response of sensors to different concentration of ammonia. Clearly, the transmission dip shifted to shorter wavelength (blue shift) with the increasing of ammonia concentration. With NH_3_ adsorbed to the sensor film, a change in the effective refractive index occurs which is measured as a wavelength shift of the interference dip. In the neutral conditions (pH = 7)^30^, the electrostatic force of the LbL multilayer membrane reaches the maximum. The strong electrostatic force leads deswelling of the film and the corresponding refractive index increases to the maximum. When the pH increases, the deprotonation reaction of PAH and PAA will happen. The charge of PAH will reduce gradually, and the charge of PAA chain will increase, which leads to the decrease of the electrostatic force between multilayer membrane and loose membrane. The negatively charged PAA will increase with increase of PH, leading to growth of the hydrophilic of membrane, so the swelling degree increases and the refractive index of film decreases. Therefore, transmission dip shifted to shorter wavelength gradually with the increasing of ammonia concentration in the process. Based on the conclusion of numerical simulation (Fig. 3b), blue shift means that the RI of the self-assembly film on the thin-core fiber Mach-Zehnder interferometer decreased. It is obvious that the experimental moving direction of dip wavelength is well consistent with the simulated one.

The data of dip wavelength *upon* concentration was fitted by a linear regression model. The result showed that the correlation coefficient R^2^ of the calibration curve was about 0.9442 (Fig. 5c) in the concentration range from 1 to 20 ppm, which indicates a good linear response of the sensor in the given concentration range. The sensitivity of the (PAH/PAA)_2_ + [PAH/(PAA + SWCNTs-COOH)]_8_ film to ammonia was estimated to be 0.031 nm/ppm.

**Figure 6:**
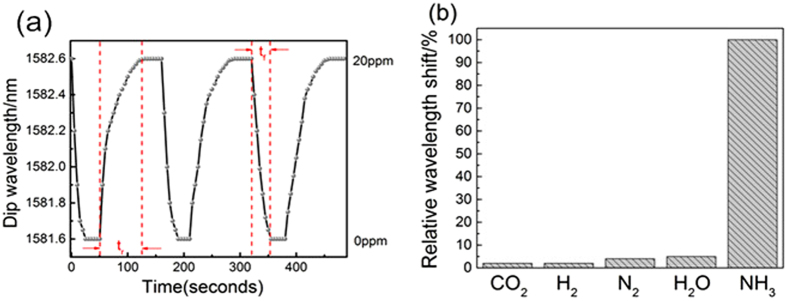
(**a**) Dynamic responses of the thin-core fiber Mach-Zehnder interferometer ammonia sensor deposited with (PAH/PAA)_2_ + [PAH/(PAA + SWCNTs-COOH)]_8_. (**b**) Relative wavelength shifts of the transmission spectrum of the TCFMI on exposure to ammonia and other analytes.

The transmission spectra were recorded every 5 seconds in order to determinate the dynamic behavior of the sensor. This measurement has been repeated several times in order to investigate the reproducibility and reversibility of the thin-core fiber Mach-Zehnder interferometer. Figure 6 shows the dynamic response of ammonia sensors coated with (PAA/PAA)_2_ + [PAH/(PAA + SWCNTs-COOH)]_8_ multilayer film place in dry N_2_ and 20 ppm ammonia gas alternatively. As shown in Fig. 6a, all the points were the peak of the transmission spectrum. The response signal could reach stable value after a spell when sensor placed in two concentration of ammonia gas alternately. The peak value changed between 1581.6 nm and 1582.6 nm showing excellent reversible and reproducible performance. Moreover, the sensor absorbs ammonia molecules rapidly but desorbs them slowly. The dynamic response rising time (t_r_) and falling time (t_f_) of the ammonia gas sensor was ca. 30 s and 75 s, respectively. This method could obtain a rapid response and reusable ammonia sensor.

The selectivity of the sensor was tested for several gases (Fig. 6b) including NH3 and common components in the atmosphere such as CO_2_, H_2_, N_2_, H_2_O. No remarkable changes were observed when the sensor was placed in the CO_2_, H_2_, H_2_O and N_2_. The sensor exhibited high sensitivity toward ammonia which shows relative wavelength shifts less than 5% of that measured in response to exposure to the ammonia gas at 100 ppm.

## Discussion

Optical fiber sensors have been investigated extensively due to light weight, immunity to electromagnetic interference, flexible design. The monitor of ammonia gas is important in many fields such as environmental and clinical analysis. Here, we have fabricated thin-core fiber Mach-Zehnder interferometer for the detection of ammonia gas. The sensor is made by splicing a small section of thin-core fiber to the standard single mode fiber. A wide-angle beam propagation method is employed for numerical simulation. The (PAH/PAA)_2_ + [PAH/(PAA + SWCNTs-COOH)]_8_ sensing film is successfully assembled on the outer surface of thin-core fiber Mach-Zehnder interferometer based on the layer-by-layer self-assembly technology. These results indicate that the characteristic wavelength shift has an approximately linear relationship with the concentration in the range from 0 ppm to 20 ppm. The shift of the dip wavelength in the experiment is consistent with the result of numerical simulation. The refraction index theoretical sensitivity is 850 nm/R.I.U. The sensor exhibited excellent selectivity, sensitivity, stability and fast response.

This work provides a facile method for fabricating multilayer films on the outer surface of thin-core fiber. The proposed thin-core fiber Mach-Zehnder interferometer sensor has advantages of simple structure, low cost and high sensitivity. It could be a candidate for environmental monitoring, chemical and clinical analysis. Future work will focus on measurement of NH_3_ at lower detection concentrations. A more rapid response involves the dispersion of CNTs and new materials to assemble the sensing film. A commercial NH_3_ sensor will be fabricated as a more accurate and efficient real-time monitoring of NH_3_ gas concentration.

## Methods

### Theory and numerical simulation

The basic principle of interferometric optical fiber sensor is as follow: The change of light’s phase is turned into the change of light’s intensity through the interferometry technology for the measurement of the change of the external physical quantity. The phase of light in the optical fiber is related to propagation constant, the geometry size of waveguide, the refractive index and the distribution of waveguide.

Modal interference means interference occurs between modes of transmission in the optical fiber, thus obtain the sensing information by analysis of interference spectrum. Modal interference includes two types: one type is that a single-mode optical fiber splicing with multimode optical fiber, the light of single-mode optical fiber coupled into the multimode optical fiber could inspire a variety of modes, interference occurs between them. The interference occurs between fundamental mode and high-order mode, considered as Mach-Zehnder interference. Another is that a single-mode optical fiber splicing with a thin-core optical fiber, or by taper dealing, laser punching, misplaced splicing and other method to makes the light transmission in single-mode optical fiber could partly coupling into the cladding, and transmitting in the cladding of optical fiber. When cladding mode coupling into fiber core of single-mode optical fiber and interfering with core mode, which is known as the optical fiber internal Mach-Zehnder interference, such as thin-core fiber Mach-Zehnder interferometer.

The sensor is based on optical fiber internal Mach-Zehnder interference, which’s interference occurs between core mode and cladding mode. Only the core model interfering with first order cladding mode, the light intensity can be expressed as:


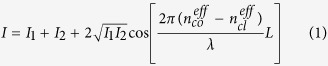


Where *I*_1_ is light intensity of core mode, *I*_2_ is light intensity of cladding mode, *λ* is free space wavelength of light in the air, *L* is the length of the optical fiber, 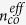 is an effective refractive index of core mode, 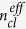 is an effective refractive index of the cladding mode. From formula (1), when the phase is the multiple of 2*π*, the intensity of interference light value reaches the minimum.


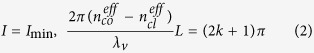


Thus, the intensity of interference light reached minimum corresponding to the wavelength as follows:


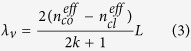


From the formula (1) (3), it can be inferred that the strength and wavelength of the interference peak related to the effective refractive index of core and cladding, length of optical fiber, etc. When parameters changing, the strength and wavelength of the interference peak will also change accordingly.

In order to investigate the performance of the sensor and carry out the optimal design of the senor, we conduct some numerical simulations. Simulations are analyzed using BeamPROP (from Rsoft Inc.), which incorporates computational techniques based on the wide-angle beam propagation method (WA-BPM).

### Materials and Experimental setup

Commercially available carboxyl modified Single-Walled Carbon nanotubes (SWCNTs-COOH, Chengdu Organic Chemicals Co.,Ltd. Chinese Academy of Sciences, Sichuan, China) having outer diameter 1–2 nm and length of 1–3 μm. Poly (allylamine hydrochloride) (PAH Mw = 65000, 20 wt% aqueous solution), poly (acrylic acid) (PAA Mw = 100 000, 35 wt% aqueous solution) were purchased from Aldrich. All of these chemicals were reagents of analytical grade, and can be used without further purification. Deionized water with a resistance of ~18 MΩ was used in all experiments.

The surface morphology of multilayer films was monitored by scanning electron microscopy (SEM) (FEI, JSM-7800F, Japan). The output interference spectra were collected and analyzed by an optical spectrum analyzer (OSA, Agilent 86140B). This configuration basically consisted of broadband light source (WYCDG-1), connected to one end of the optical fiber in order to couple the light into the optical fiber. All the measurements were performed at room conditions (25 °C).

### Fabrication of the thin-core fiber modal interferometer

SWCNTs-COOH and PAA (1:1 wt/wt) were dispersed in deionized water to give a total concentration of 2 mg/mL. PAH and PAA were diluted to 2 mg/mL by deionized water respectively. A thin-core fiber modal interferometer was fabricated by fusion splicing a section of thin-core fiber (TCF, length of 2 cm, core diameter 2.5 μm, Nufern 460-HP) in between two single mode fibers (SMF-28e, Corning).

Then the thin-core fiber Mach-Zehnder interferometer was cleaned with acetone and ethanol, respectively. After that interferometer was immersed in freshly prepared piranha solution (3:7 v/v mixture of 30% H_2_O_2_ and concentrated H_2_SO_4_) at 90 °C for 120 min, followed by thoroughly rinsing with large amount of deionized water, and then dried with ultra high pure nitrogen. The surface of optical fiber was negatively charged. The activated interferometer was immersed into the PAH and PAA solutions alternatively for 10 min to form the function film through electrostatic adsorption. After each film was formed, the interferometer was rinsed for three times by deionized water to remove the excess molecules and dried with nitrogen. Repeating the step twice, a (PAH/PAA)_2_ multilayer precursor film was deposited on the surface of interferometer. It could increase the charge density, which is beneficial to the adsorption of SWCNTs-COOH. Then the interferometer was immersed into the PAH and PAA + SWCNTs-COOH blended solution alternatively to form the function film. Repeating this step, (PAH/PAA)_2_ + [PAH/(PAA + SWCNTs-COOH)]_8_ multilayer film was deposited on the surface of interferometer. Finally, the interferometer was dried under 60 °C for 10 h before sensing tests and characterized by scanning electronic microscope.

## Additional Information

**How to cite this article**: Huang, X. *et al*. An in-line Mach-Zehnder Interferometer Using Thin-core Fiber for Ammonia Gas Sensing With High Sensitivity. *Sci. Rep.* **7**, 44994; doi: 10.1038/srep44994 (2017).

**Publisher's note:** Springer Nature remains neutral with regard to jurisdictional claims in published maps and institutional affiliations.

## Figures and Tables

**Table 1:** Parameters of optical fiber used in simulation.

Items	SMF	TCF
**Diameter of core (μm)**	8.3	2.5
**Refractive index of core**	1.4512	1.4505
**Diameter of cladding (μm)**	125	125
**Refractive index of cladding**	1.4447	1.4447
